# A new species, *Aster yaoshanensis* (Asteracae, Astereae), from Guangxi (China), based on morphology and molecular phylogenetic data

**DOI:** 10.3389/fpls.2024.1367917

**Published:** 2024-04-02

**Authors:** Xinyi Zheng, Kun Qin, Tingyu Li, Tianmeng Qu, Junjia Luo, Guojin Zhang, Bo Li, Pan Li, Zhixi Fu

**Affiliations:** ^1^ Key Laboratory of Land Resources Evaluation and Monitoring in Southwest, Sichuan Normal University, Ministry of Education, Chengdu, China; ^2^ College of Life Sciences, Sichuan Normal University, Chengdu, China; ^3^ Department of Protection, Dayaoshan Mountain National Nature Reserve, Laibin, China; ^4^ College of Life Sciences, Hunan Normal University, Changsha, China; ^5^ Sichuan Environmental Monitoring Center, Chengdu, China; ^6^ College of Life Sciences, Zhejiang University, Hangzhou, China; ^7^ Sustainable Development Research Center of Resources and Environment of Western Sichuan, Sichuan Normal University, Chengdu, China

**Keywords:** Asteraceae, *Aster*, Guangxi, new species, phylogeny

## Abstract

*Aster yaoshanensis* sp. nov., a new species of the family Asteraceae is here described and illustrated. The species is presently known only from rock crevices of mountain valleys in Dayaoshan National Nature Reserve, Guangxi autonomous region, China. Phylogenetic analyses based on ITS sequences and complete plastome data have shown that this new species is a member of genus *Aster* with high support. Morphologically, it mostly resembles *A. jishouensis*, but it can be easily distinguished from the latter by bract indumentum (glabrous except margin ciliate vs. villous especially on veins abaxially, glabrous adaxially) and color (green vs. purple), shorter corolla (3.2–3.5 mm vs. 4.5–5.3 mm), bract stalk (obvious, ca.1.2 mm vs. sessile), and different distribution (Guangxi vs. Hunan). The detailed description, distribution map, and photos are provided. This study further elucidates the species identification, phylogeny and characteristic evolution of *Aster*.

## 1 Introduction

The genus *Aster* with about 152 species is one of the largest genera in tribe Astereae (Asteraceae) (Nesom, 1994; Noyes and Rieseberg, 1999; Nesom and Robinson, 2007; Brouillet et al., 2009; Chen et al., 2011; Fu et al., 2016). So far, 123 species of *Aster* have been identified from different regions of China (Chen et al., 2011; Xiong et al., 2019). To revise the genus *Aster*, we have conducted extensive collections and observations in the field in Eurasia. In Guangxi province of China, Asteraceae is represented by 81 genera and 233 species, and the genus *Aster* includes 15 species (Wei, 2018).

On 3 November 2020, Dr. Pan Li (Zhejiang University) collected some specimens of a species belonging to genus *Aster* in Dayaoshan National Nature Reserve, Jinxiu county, Laibin city, Guangxi province, China. Then, in 12 November 2020, Dr. Zhixi Fu revisited and collected the distinctive species in the same location. Through field investigation and morphological study, we confirmed that this species does not match any previously published description from southwest China to central Himalaya. Further molecular analyses revealed significant differences between this species and its relatives. The results allow us to infer that these newly collected specimens from Guangxi belong to a new species.

High rates of morphological divergence are a challenge to morphology-based classification, because some of this variation originated by parallel evolution whereas other characters are apomorphic (Wei et al., 2013). In previous studies, the reconstruction of the ancestral state of morphological characteristics has confirmed the hypothesis on the origin and evolution of the morphological diversity of some species in Asteraceae (Norbert et al., 2017). There are still insufficient studies on the character evolution of *Aster*, as in other Asteraceae genera with many species and a complex taxonomy (Wagensommer et al., 2016). Based on the ancestral reconstruction, we analyzed some morphological traits of the new species and its relatives in *Aster*.

In the context of the ongoing taxonomic revision of the genus *Aster*, this finding allows us to more precisely define its morphological characters, complete plastome characters, phylogenetic position, taxonomic affinities, and ancestral trait. Therefore, the present work aims (1) to provide a complete morphological record of the new species and distribution map; (2) to examine the phylogenetic affinities of *A. yaoshanensis* and its related species using the complete plastome sequences and nuclear ribosomal DNA internal transcribed spacer (ITS) sequences; and (3) to provide hypotheses for the evolution of morphological characters of *A. yaoshanensis* and by integrating available information, to provide an adequate taxonomic treatment for this new species.

## 2 Materials and methods

### 2.1 Sample collection, DNA extraction, PCR reaction, and sequencing

Our taxon sampling strategy was designed to include *Aster* and relatives genera based on the recent classification (Li et al., 2012; Fu et al., 2016). We downloaded nrDNA ITS sequence matrix of 71 species from other molecular phylogenetic studies (Li et al., 2012; Zhang et al., 2015a; Zhang et al., 2019b), representing 17 genera and major clades of *Aster* and its relatives. Meanwhile, we downloaded the complete plastome sequence matrix of 17 species, representing the genus *Aster* and its relatives.

One fresh leave for each specimen was dried in silica gel (the list of samples is shown in **Table 1**). Total genomic DNA was extracted using the Plant Genomic DNA Kit (Tiangen Biotech, Beijing, China) following the CTAB DNA extraction protocol (Doyle and Doyle, 1987) and the manufacturer’s instructions. In this study, to determine the systematic position of the new species, we used nuclear ribosomal DNA ITS sequences for phylogenetic inference. The spacer region of the nuclear ribosomal repeat (nrDNA ITS) was separately amplified with standard PCR following the method described in Zhang et al. (2015, 2019). The new species, *A. yaoshanensis* was sequenced, and the GenBank accession numbers and sample information of the ITS sequences used in this study are shown in **Table 2** (accession number of *A. yaoshanensis*: OL461705.1). Voucher specimens were deposited at the herbarium of the Sichuan Normal University (SCNU).

**Table 1:** The list of the collected samples of *A. yaoshanensis* in our study.

Locality	Collection date	Collectors	Herbarium code
Jinxiu, Guangxi	12-Nov-2020	*K. Qin & Z.X. Fu*	*FZX5501* (SCNU)
Jinxiu, Guangxi	12-Nov-2020	*K. Qin & Z.X. Fu*	*FZX5505* (SCNU)
Jinxiu, Guangxi	12-Nov-2020	*K. Qin, & Z.X. Fu*	*FZX5508* (SCNU)
Jinxiu, Guangxi	12-Nov-2020	*K. Qin & Z.X. Fu*	*FZX5509* (SCNU)
Jinxiu, Guangxi	12-Nov-2020	*K. Qin & Z.X. Fu*	*FZX5510* (SCNU)
Jinxiu, Guangxi	12-Nov-2020	*K. Qin & Z.X. Fu*	*FZX5511* (SCNU)

**Table 2:** Taxa sampled and their GenBank accession numbers for the ITS sequences used in this study.

Taxon	Voucher and reference	GenBank accessions ITS
*Arctogeron gramineum* (L.) DC.	Li et al. (2012) *LWP0606014* (HNNU)	JN315928
*Aster ageratoides* var. *lasiocladus* (Hayata) Hand.-Mazz.	Li et al. (2012) *LWP0112018* (HNNU)	JN543781
*Aster albescence* var. *albescens*	Li et al. (2012) *LWP0508123* (HNNU)	JN543862
*Aster alpinus* L.	Li et al. (2012) *LWP0607020* (HNNU)	JN543817
*Aster amellus* L.	Li et al. (2012) *LWP0408002* (HNNU)	JN543742
*Aster argyropholis* Hand.-Mazz.	Li et al. (2012) *LWP0409045* (HNNU)	JN543793
*Aster asteroides* (DC.) O. Ktze.	Li et al. (2012) *LWP0708112* (HNNU)	JN543841
*Aster auriculatus* Franch.	Li et al. (2012) *LWP0509059* (HNNU)	JN543754
*Aster baccharoides* (Benth.) Steetz.	Li et al. (2012) *LWP0802001*(HNNU)	JN543805
*Aster batangensis* Bureau & Franch.	Li et al. (2012) *LWP0606039* (HNNU)	JN543859
*Aster brachytrichus* Franch.	Li et al. (2012) *LWP0607075* (HNNU)	JN543838
*Aster diplostephioides* (DC.) C.B.Clarke	Li et al. (2012) *LWP0507020* (HNNU)	JN543847
*Aster dolichopodus* Y. Ling	Li et al. (2012) *LWP0409060* (HNNU)	JN543775
*Aster falcifolius* Hand.-Mazz.	Li et al. (2012) *LWP0410050* (HNNU)	JN543802
*Aster fanjingshanicus* Y.L. Chen & D.J. Liu	Li et al. (2012) *LWP0606082* (HNNU)	JN543829
*Aster flaccidus* Bunge	Li et al. (2012) *LWP0607026*(HNNU)	JN543844
*Aster fuscescens* Bur. & Franch.	Li et al. (2012) *YGS1007021* (HNNU)	JN543751
*Aster handelii* Onno	Li et al. (2012) *LWP0708174* (HNNU)	JN543820
*Aster hersileoides* C.K.Schneid.	Li et al. (2012) *LWP0807002* (HNNU)	JN543787
*Aster heterolepis* Hand.-Mazz.	Li et al. (2012) *LWP0507004* (HNNU)	JN543823
*Aster homochlamydeus* Hand.-Mazz.	Li et al. (2012) *LWP0508004* (HNNU)	JN543784
*Aster jishouensis* W.P. Li & S.X. Liu	Li et al. (2012) *LWP1012015* (HNNU)	JN543808
*Aster lavanduliifolius* Hand.-Mazz.	Li et al. (2012) *LWP0708053* (HNNU)	JN543796
*Aster maackii* Regel	Li et al. (2012) *LWP0609043* (HNNU)	JN543745
*Aster mangshanensis* Ling	Li et al. (2012) *LWP0511034* (HNNU)	JN543760
*Aster nitidus* Chang	Li et al. (2012) *LWP0505007* (HNNU)	JN543790
*Aster oreophilus* Franch.	Li et al. (2012) *LWP0509016* (HNNU)	JN543826
*Aster panduratus* Nees ex Walper	Li et al. (2012) *LWP1012067* (HNNU)	JN543757
*Aster poliothamnus* Diels	Li et al. (2012) *LWP0506001* (HNNU)	JN543763
*Aster pycnophyllus* W.W. Smith	Li et al. (2012) *LWP0509091* (HNNU)	JN543799
*Aster sampsonii* (Hance) Hemsl.	Li et al. (2012) *LWP0511060* (HNNU)	JN543811
*Aster senecioides* Franch.	Li et al. (2012) *LWP0708215* (HNNU)	JN543856
*Aster sikuensis* W.W. Smith et Farr.	Li et al. (2012) *LWP0510025* (HNNU)	JN543766
*Aster smithianus* Hand.-Mazz.	Li et al. (2012) *LWP0508034* (HNNU)	JN543778
*Aster souliei* Franch.	Li et al. (2012) *LWP0708084* (HNNU)	JN543835
*Aster sutchuenensis* Franch.	Li et al. (2012) *LWP0508007* (HNNU)	JN543850
*Aster taliangshanensis* Ling	Li et al. (2012) *LWP0607056* (HNNU)	JN543772
*Aster tataricus* L. f.	Li et al. (2012) *LWP0108018* (HNNU)	JN543748
*Aster tongolensis* Franch.	Li et al. (2012) *LWP0708147* (HNNU)	JN543832
*Aster turbinatus* S. Moore	Li et al. (2012) *LWP0110029* (HNNU)	JN543814
*Aster vestitus* Franch.	Li et al. (2012) *LWP0509023* (HNNU)	JN543769
*Aster yunnanensis* Franch.	Li et al. (2012) *LWP0508089* (HNNU)	JN543853
*Asterothamnus centraliasiaticus* Novopokr.	Li et al. (2012) *LWP0607045* (HNNU)	JN315930
*Asterothamnus fruticosus* (C. Winkl.) Novopokr.	Li et al. (2012) *LWP0607005* (HNNU)	JN315929
*Callistephus chinensis*(L.) Nees	Li et al. (2012) *LWP0108021* (HNNU)	JN315931
*Chrysanthemum coronarium* L.	Li et al. (2012) *LWP1004010* (HNNU)	JN315939
*Conyza japonica* (Thunb.) Less.	Li et al. (2012) *LWP0606032* (HNNU)	JN315938
*Conyza sumatrensis* (S.F. Blake) Pruski & G. Sancho	Li et al. (2012) *LWP1009002* (HNNU)	JN315923
*Dendranthema indicum*(L.) Des Moul.	Li et al. (2012) *LWP1012002* (HNNU)	JN315940
*Doellingeria scaber* (Thunb.) Nees	Li et al. (2012) *LWP0108025* (HNNU)	JN315934
*Doellingeria umbellata*	Li et al. (2012)	AF046966
*Erigeron annus* (L.) Pers.	Li et al. (2012) *LWP1010009* (HNNU)	JN315924
*Erigeron breviscapus*	Li et al. (2012) *LWP0606055* (HNNU)	JN315925
*Galatella dahurica* DC.	Li et al. (2012) *LWP0609047* (HNNU)	JN315935
*Grangea maderaspatana* (L.) Poir.	Li et al. (2012) *LWP0802034* (HNNU)	JN315920
*Heteropappus altaicus* var. *millefolius*	Li et al. (2012) *LWP0506010* (HNNU)	JN543709
*Heteropappus crenatifolius* (Hand.-Mazz.) Griers.	Li et al. (2012) *LWP0409037* (HNNU)	JN543712
*Kalimeris incisa* (Fisch.) DC.	Li et al. (2012) *LWP0609107* (HNNU)	JN543721
*Kalimeris indica* (L.) Sch.-Bip.	Li et al. (2012) *LWP0806017* (HNNU)	JN543715
*Kalimeris integrifolia* Turcz. ex DC.	Li et al. (2012) *LWP0609077* (HNNU)	JN543718
*Kalimeris longipetiolata* (Chang) Ling	Li et al. (2012) *LWP0508104* (HNNU)	JN315936
*Miyamayomena angustifolius*	Li et al. (2012) *DBY9206* (HNNU)	JN543736
*Miyamayomena piccolii* (J.D. Hooker) Kitamura	Li et al. (2012) *LWP0510055* (HNNU)	JN543730
*Myriactis nepalensis* Less.	Li et al. (2012) *LWP0509002* (HNNU)	JN315921
*Myriactis wightii* DC.	Li et al. (2012) *LWP0509010* (HNNU)	JN315922
*Rhinactinidia eremophila* (Bunge) Novopokrovsky ex Botschantzev	Li et al. (2012) *LWP0607036* (HNNU)	JN543727
*Rhinactinidia limoniifolia* (Lessing) Novopokrovsky ex Botschantzev	Li et al. (2012) *LWP0607012* (HNNU)	JN543724
*Rhynchospermum verticillatum*Reinw.	Li et al. (2012) *LWP0607065* (HNNU)	JN543706
*Sheareria nana* S. Moore	Li et al. (2012) *LWP0701001* (HNNU)	JN543703
*Tripolium vulgare* Nees	Li et al. (2012) *LWP0311001* (HNNU)	JN315937
*Turczaninowia fastigiata* (Fisch.) DC.	Li et al. (2012) *LWP0609030* (HNNU)	JN543739
***Aster yaoshanensis***	This study, *FZX5501* (SCNU)	**OL461705**

In this study, we also used the complete plastome sequence of *A. yaoshanensis* for phylogenetic inference. We followed the CTAB DNA extraction protocol (Doyle and Doyle, 1987) and the manufacturer’s instructions, to extract total genomic DNA of *A. yaoshanensis*, using the Plant Genomic DNA Kit (Tiangen Biotech, Beijing, China). Then, the NanoDrop 2000 Spectrophotometer and Qubit 4 Fluorometer (Thermo Fisher Scientific, Wilmington, DE, USA) were used to test quality and quantity of the total genomic DNA. We constructed DNA libraries using Illumina Paired-End DNA Library Kit (Illumina Inc., San Diego, CA, USA) in the NovaSeq 6000 platform with a paired-end reading length of 150 bp (NovoGene Inc., Beijing, China).

### 2.2 Plastome assembly, annotation, and feature analyses

The complete plastome of *A. yaoshanensis* was assembled by SPAdes3.15.1 (Bankevich et al., 2012) with default parameters. The circular plastome was obtained by using Bandage (Wick et al., 2015). Subsequently, the results were annotated using PGA (Qu et al., 2019). The annotation results were checked using Geneious R11 (Kearse et al., 2012). The plastome map was drawn on the OGDRAW (https://chlorobox.mpimp-golm.mpg.de/OGDraw.html). The tRNA sequences were also confirmed by tRNAscan-SE v2.0.7 (Lowe and Eddy, 1997), on the platform Geseq (https://chlorobox.mpimp-golm.mpg.de/geseq.html). The genome sequence of *A. yaoshanensis* has been deposited in GenBank (accession number: ON120847.1). We performed nucleobase content and genes analysis using the platform JSHYClound (www.jshycloud.net).

### 2.3 Phylogenetic reconstruction of the ITS data set

Phylogenetic analyses of the ITS data set (**Table 2**) were performed using maximum likelihood (ML) and Bayesian inference (BI) analysis using RAxML (Stamatakis et al., 2008) and MrBayes (Ronquist et al., 2012), respectively, on the CIPRES science gateway portal (https://www.phylo.org/portal2/, Miller et al., 2010). Prior to ML and BI analyses, the best fit model selected was GTR+T+G according to the Akaike information criterion (AIC) implemented in ModelTest (Posada and Crandall, 1998). RAxML was conducted with the fast bootstrap option, using 1,000 replicates, bootstrap value (BS) of 80%–100% were interpreted as strong support, 60%–80% as moderate. In BI analysis, four Markov chain Monte Carlo chains were run for 7,000,000 generations each, and were sampled every 1,000 generations, starting with a random tree, and Bayesian posterior probabilities (PP) were calculated for the majority consensus tree of all sampled trees after discarding 25% of trees sampled. Bayesian PP more than 0.95 were considered to be strong support, from 0.9 to 0.95 to be moderate. Finally, the trees were visualized using FigTree (Rambaut, 2012). The species *Chrysanthemum indicum* L. and *Dendranthema indicum* (L.) Des Moul. were selected as the outgroup following previous works (Panero and Funk, 2002, 2008; Panero et al., 2014).

### 2.4 Phylogenetic reconstruction of the complete plastsome sequence data set

To investigate the phylogenetic positions of *A. yaoshanensis* within the *Aster* genus, 17 complete plastome sequences were downloaded from the NCBI database (**Table 3**). Phylogenetic analyses of the complete plastome data set were performed using ML in RAxML on the CIPRES platform (https://www.phylo.org/portal2/). We used ModelTest to predict the best fit model. The fast bootstrap option and 1,000 replicates were used in RAxML. Finally, the trees were visualized using FigTree.

**Table 3:** Taxa sampled and their GenBank accession numbers for the whole plastome sequences used in this study.

Taxon	GenBank accession numbers
*Aster souliei*	OK323961.1
*Aster tongolensis*	OK323962.1
*Aster altaicus*	NC034996
*Aster pekinensis*	MW255593
*Aster tataricus*	NC042913
*Aster indicus*	MG710386.1
*Aster ageratoides* var. *scaberulus*	MW813970
***Aster yaoshanensis***	**ON120847**
*Heteroplexis incana*	NC048508.1
*Heteroplexis sericophylla*	MK942054.1
*Aster hypoleucus*	NC046503
*Aster hersileoides*	NC042944
*Symphyotrichum subulatum*	NC050667
*Erigeron Canadensis*	MT806101.1
*Aster batangensis*	MZ292735.1
*Aster flaccidus*	MN122101
*Llerasia caucana*	NC034821.1
*Nannoglottis ravida*	NC053322.1

### 2.5 Ancestral trait reconstruction

Based on the topology of the ITS sequence for ML analysis, in Mesquite v3.81 (Maddison, 2008), we used the “Trace character history” option to analyze the ancestral morphological characters in the genus *Aster*. An ML approach using Markov k-state 1 parameter model (Mk1; Lewis, 2001) was used to reconstruct character evolution. The data employed for the reconstruction of the evolution of morphological characters were obtained from literatures (Li et al., 2012; Norbert et al., 2017), and our own observations of morphological character variation using herbarium specimens held at SCNU and PE (Thiers, 2024), and CVH (https://www.cvh.ac.cn/). The codes associated with these morphological features include (A) Inflorescence type: listed in order of 0–7, (B) the length of pappus: (0) none pappus, (1) short pappus, (2) long pappus, (3) longer pappus; (C) habit (0) dwarf herb, (1) tall herb, (2) subshrub, (3) shrub. A list of morphological characters and their character state coding used for the ancestral state reconstruction is detailed in **Table 4**. The differences of eight types of inflorescence can be seen in the statement at the end of **Table 4**. Based on the codes, we reconstructed the evolution of three morphological characters for genera of *Aster* and its relatives.

**Table 4:** Diagnostic feature coding used to infer the Bayesian stochastic character mapping analyses.

Taxon	Habit	Pappus length	Inflorescence
*Arctogeron gramineum* (L.) DC.	0	3	0
*Aster ageratoide*s var. *lasiocladus* (Hayata) Hand.-Mazz.	1	3	2
*Aster albescens*	3	3	2
*Aster alpinus* L.	0	3	0
*Aster amellus* L	0	3	1
*Aster argyropholis* Hand.-Mazz.	3	3	2
*Aster asteroides* (DC.) O. Ktze.	0	3	0
*Aster auriculatus* Franch.	1	3	2
*Aster baccharoides* (Benth.) Steetz.	2	3	2
*Aster batangensis* Bureau & Franch.	2	3	0
*Aster brachytrichus* Franch.	0	2	0
*Aster diplostephioides* (DC.) C.B.Clarke	0	3	0
*Aster dolichopodus* Y. Ling	1	3	1
*Aster falcifolius* Hand.-Mazz.	0	3	4
*Aster fanjingshanicus* Y.L. Chen & D.J. Liu	0	3	0
*Aster flaccidus* Bunge	0	3	0
*Aster fuscescens* Bur. & Franch.	1	3	2
*Aster handelii* Onno	0	3	0
*Aster hersileoides* C.K.Schneid.	3	3	3
*Aster heterolepis* Hand.-Mazz.	1	3	1
*Aster homochlamydeus* Hand.-Mazz.	1	3	2
*Aster jishouensis W*.P. Li & S.X. Liu	1	3	5
*Aster lavandulifolius* Hand.-Mazz.	3	3	2
*Aster maackii* Regel	1	3	1
*Aster mangshanensis* Ling	1	3	2
*Aster nitidus* Chang	3	3	2
*Aster oreophilus* Franch.	0	3	1
*Aster panduratus* Nees ex Walper	1	3	2
*Aster poliothamnus* Diels	2	3	1
*Aster pycnophyllus* W.W. Smith	1	3	2
*Aster sampsonii* (Hance) Hemsl.	1	2	2
*Aster senecioides* Franch.	1	3	2
*Aster sikuensis* W.W. Smith et Farr.	2	3	1
*Aster smithianus* Hand.-Mazz.	2	2	2
*Aster souliei* Franch.	0	2	0
*Aster setchuenensis* Franch.	1	3	1
*Aster taliangshanensis* Ling	1	3	0
*Aster tataricus* L. f.	1	3	2
*Aster tongolensis* Franch.	1	2	0
*Aster turbinatus* S. Moore	1	3	1
*Aster vestitus* Franch.	1	3	2
*Aster yunnanensis* Franch.	1	3	0
*Asterothamnus centraliasiaticus* Novopokr.	2	3	6
*Asterothamnus fruticosus* (C. Winkl.) Novopokr.	2	3	6
*Callistephus chinensis*(L.) Nees	1	3	0
*Chrysanthemum coronarium* L.	1	3	1
*Conyza japonica* (Thunb.) Less.	1	3	2
*Conyza sumatrensis* (S.F. Blake) Pruski & G. Sancho	1	3	2
*Dendranthema indicum*(L.) Des Moul.	1	0	2
*Doellingeria scaber* (Thunb.) Neesb.	1	3	2
*Doellingeria umbellata* (Mill.) Nees	1	3	2
*Erigeron annus* (L.) Pers.	1	3	2
Erigeron *breviscapus* (Vant.) Hand.-Mazz.	1	3	0
*Galatella dahurica* DC.	1	3	1
*Grangea maderaspatana* (L.) Poir.	0	0	1
*Heteropappus altaicus* var. *millefolius*	1	3	1
*Heteropappus crenatifolius* (Hand.-Mazz.)	1	3	1
*Kalimeris incisa* (Fisch.) DC.	1	1	1
*Kalimeris indica* (L.) Sch.-Bip.	1	1	1
*Kalimeris integrifolia* Turcz. ex DC.	1	1	1
*Kalimeris longipetiolata* (Chang) Ling	1	1	1
*Aster angustifolius*	1	0	1
*Miyamayomena piccolii* (J.D. Hooker) Kitamura.	1	0	2
*Myriactis nepalensis* Less.	1	0	1
*Myriactis wightii* DC.	1	0	2
*Rhinactinidia eremophila*	0	3	0
*Rhinactinidia limoniifolia* (Lessing) Novopokrovsky ex Botschantzev	0	3	0
*Rhynchospermum verticillatum* Reinw.	1	3	2
*Sheareria nana* S. Moore	1	0	2
*Tripolium vulgare* Nees	1	3	2
*Turczaninowia fastigiata* (Fisch.) DC.	1	3	2
*Aster yaoshanensis*	1	3	7

The feature coding of inflorescence type include: (0) Capitula solitary at ends of scapiform stems. (1) Stems erect, capitula 1-10 in terminal racemiform synflorescences. (2) Stems multibranched, capitula 7-38 in terminal, open corymbiform synflorescences. (3) *Aster hersileoides*: stems many branched, old branches erect, procumbent or ascending, capitula at ends of second-year axillary branches, solitary. (4) *Aster falcifolius*: stems erect, capitula in terminal or axillary, sessile or peduncles 0.1-20 mm. (5) Caulis adscendens, capitula radiata, 1-4 ad apices ramorum vel in axillis. (6) Subshrubs, rhizome thick, stems numerous, erect or ascending, many branched in lower part. (7) Caulis adscendens, capitula numerous, at axillary branches. These types correspond to the legend of **Figure 6** from top to bottom.

### 2.6 Conservation status

With the information collected through fieldwork, the conservation status of the new species was evaluated according to the IUCN, 2024 categories and criteria.

## 3 Results

### 3.1 Plastome genome features

**Figure 1:**
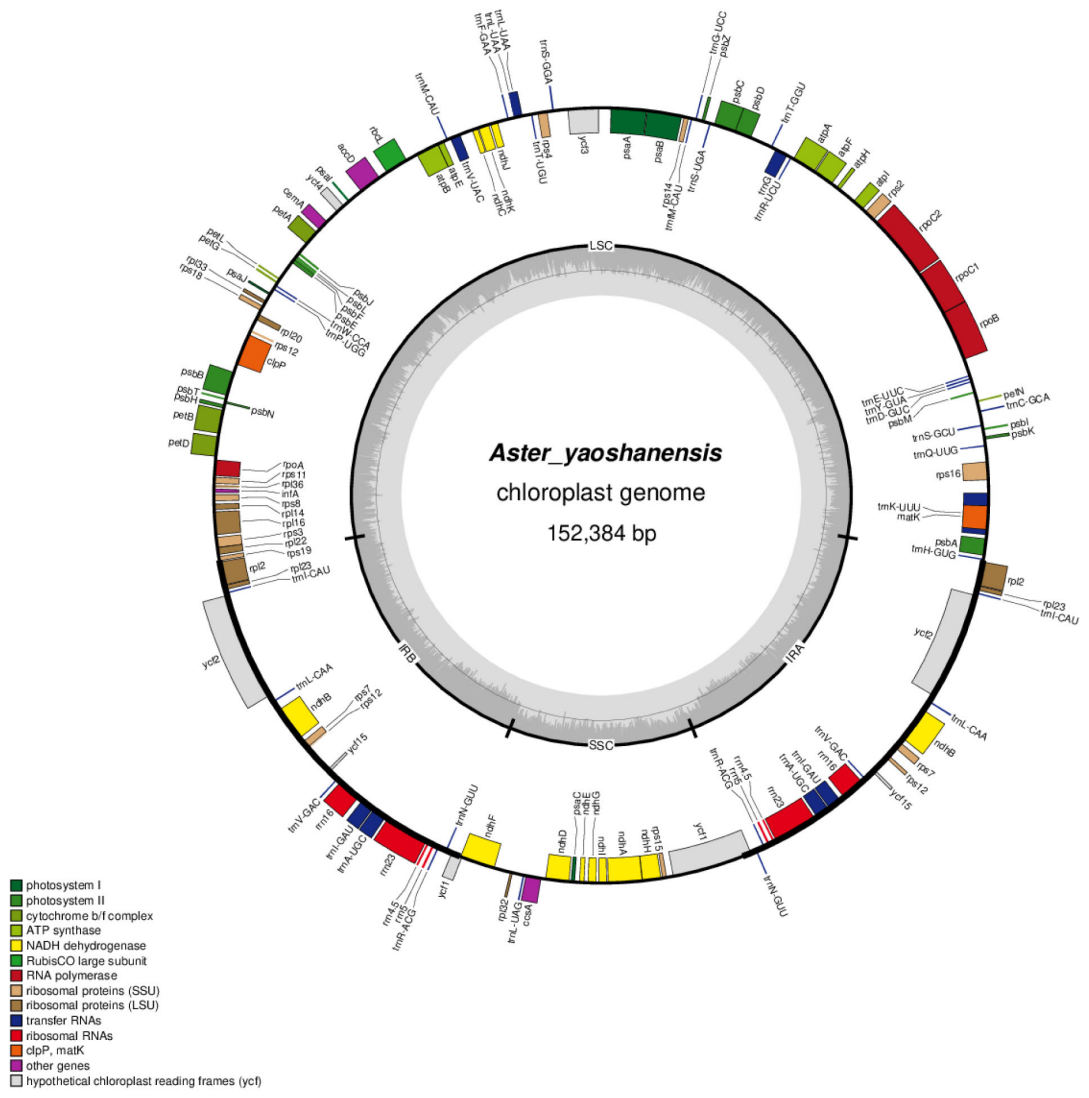
Gene maps of the plastomes of *A. yaoshanensis*.

In this study, the structure of plastome of *A. yaoshanensis* is highly conserved, and it has standard quadripartite structure, including a large single-copy (LSC), a small single-copy (SSC), and a pair of IRs (IRa and IRb). The size o f *A. yaoshanensis* is 152,384bp, and GC content is 37.37%. Overall, the plastome have 133 genes, including 87 protein-coding genes, 38 tRNA, and 8 rRNA, of which 115 were unique and 18 were duplicated in the IR regions (**Table 5**). The arrangements of these 133 genes were totally collinear. There are two introns of three genes (*rps*12, *ycf*3, *clp*P), and single intron of 16 genes (*ndh*A, *ndh*B*, pet*B*, pet*D*, atp*F*, rbc*L*, rpl*16*, rpl*2*, rps*16*, rpo*C1*, trn*A-UGC*, trn*G*, trn*I-GAU*, trn*K-UUU*, trn*L-UAA*, trn*V-UAC). The gene rps12 was trans-spliced and the genes *ndh*D, *psb*L were RNA editing (**Table 5**). These results were performed in the plastome map (**Figure 1**).

**Table 5:** List of genes found in the complete plastomes of *Aster* species.

Category	Gene group	Gene name
Photosynthesis	Subunits of photosystem I	*psa*A, *psa*B, *psa*C, *psa*I, *psa*J
	Subunits of photosystem II	*psb*A, *psb*B, *psb*C, *psb*D, *psb*E, *psb*F, *psb*H, *psb*I, *psb*J, *psb*K, *psb*L, *psb*M, *psb*N, *psb*T, *psb*Z
	Subunits of NADH dehydrogenase	*ndh*A*, *ndh*B*(2), *ndh*C, *ndh*D, *ndh*E, *ndh*F, *ndh*G, *ndh*H, *ndh*I, *ndh*J, *ndh*K
	Subunits of cytochrome b/f complex	*pet*A, *pet*B*, *pet*D*, *pet*G, *pet*L, *pet*N
	Subunits of ATP synthase	*atp*A, *atp*B, *atp*E, *atp*F*, *atp*H, *atp*I
	Large subunit of rubisco	*rbc*L*
Self-replication	Subunits of RNA polymerase	*rpo*A, *rpo*B, *rpo*C1*, *rpo*C2
	Ribosomal RNAs	*rrn*4.5(2), *rrn*5(2), *rrn*16(2), *rrn*23(2)
	Proteins of large ribosomal subunit	*rpl*14, *rpl*20, *rpl*22, *rpl*23(2), *rpl*16*, *rpl*2*(2), *rpl*32, *rpl*33, *rpl*36
	Proteins of small ribosomal subunit	*rps*11*, rps*12**(2), *rps*14, *rps*15, *rps*16**, rps18, *rps*19, *rps*2, *rps*3, *rps*4, *rps*7(2), *rps*8
	Transfer RNAs	*trn*A-UGC*(2)*, trn*C-GCA, *trn*D-GUC, *trn*E-UUC, *trn*F-GAA, *trn*G*, *trn*G-UCC, *trn*H-GUG, *trn*I-CAU(2), *trn*I-GAU*(2), *trn*K-UUU*, *trn*L-CAA(2), *trn*L-UAA, *trn*L-UAA*, *trn*L-UAG, *trn*M-CAU, *trn*N-GUU(2), t*rn*P-UGG, *trn*Q-UUG, *trn*R-ACG(2), *trn*R-UCU, *trn*S-GCU, *trn*S-GGA, *trn*S-UGA, *trn*T-GGU, *trn*T-UGU, *trn*V-GAC(2), *trn*V-UAC*, *trn*W-CCA, *trn*Y-GUA, *trnf*M-CAU
Other genes	Protease	*clp*P**
	Maturase	*mat*K
	Envelope membrane protein	*cem*A
	Translation initiation factor	*inf*A
	c-type cytochrome synthesis gene	*ccs*A
	Acetyl-CoA carboxylase	*acc*D
Genes of unknown function	Conserved hypothetical chloroplast ORF	*#ycf*1*, ycf*1*, ycf*2(2)*, ycf*3**, ycf4*, ycf*15(2)

Gene*: Gene with one introns; Gene**: Gene with two introns; #Gene: Pseudo gene; Gene(2): number of copies of multi-copy genes.

### 3.2 Phylogenetic analysis of the ITS data set

**Figure 2:**
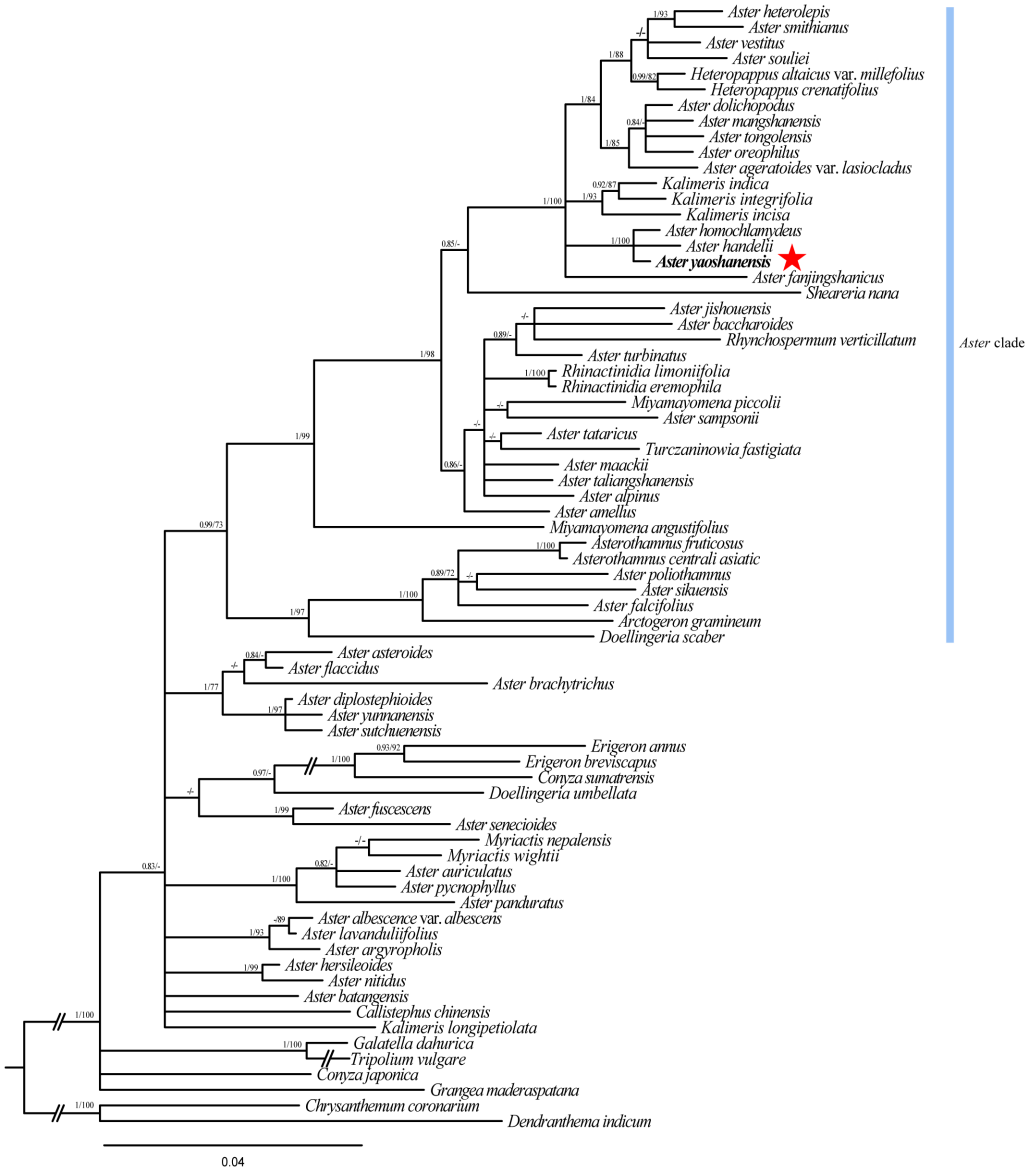
Cladogram of the Beyasian *i*nference (BI) phylogenetic tree of *Aster*. Phylogenetic tree based on nrDNA data (ITS), showing the position of *A. yaoshanensis* (in red star). Values above branch represent Bayesian posterior probabilities (PP) and bootstrap values (BS), respectively; bootstrap support values >0.80 (BI) or >60% (ML) are shown. Blue represents *Aster* clade.

The total length of ITS sequence alignment with gaps was 650 bp. Analysis of the data using ML and BI methods obtained similar trees with high ML Bootstrap (BS) and BI posterior probability (PP). Phylogeny analysis showed that *A. yaoshanensis* is positioned in the *Aster* clade, and it formed a clade with *A. homochlamydeus* Hand.-Mazz and *A. handelii* Onno (ML BS ≥ 90% and BI PP ≥ 0.90) (**Figure 2**).

### 3.3 Phylogenetic reconstruction of the complete plastome sequence data set

**Figure 3:**
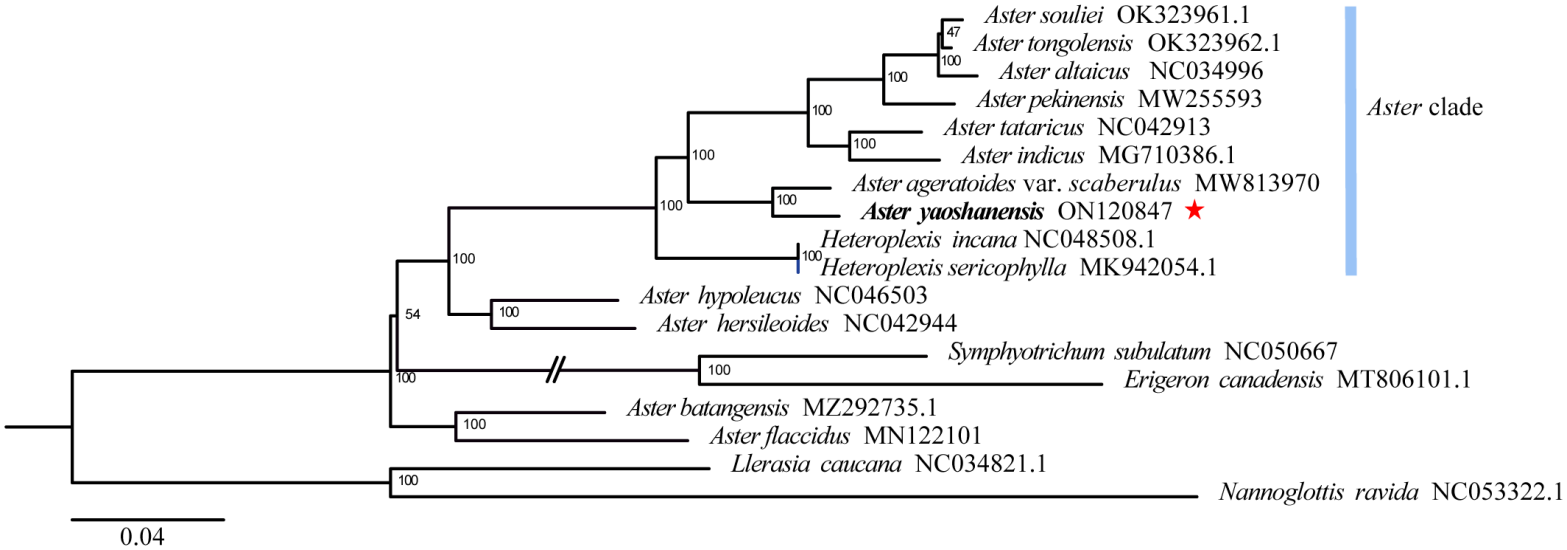
Maximum likelihood (ML) phylogenetic tree based on complete plastome sequences, showing the position of *A. yaoshanensis* (in red star). Blue represent *Aster* clade.

Based on phylogenetic analysis, *A. yaoshanensis* is positioned in the *Aster* clade, and it is the sister species to *A. ageratoides* var. *scaberulus* (Miq.) Y. Ling. (BS = 100) (**Figure 3**).

### 3.4 Morphological analysis

**Figure 4:**
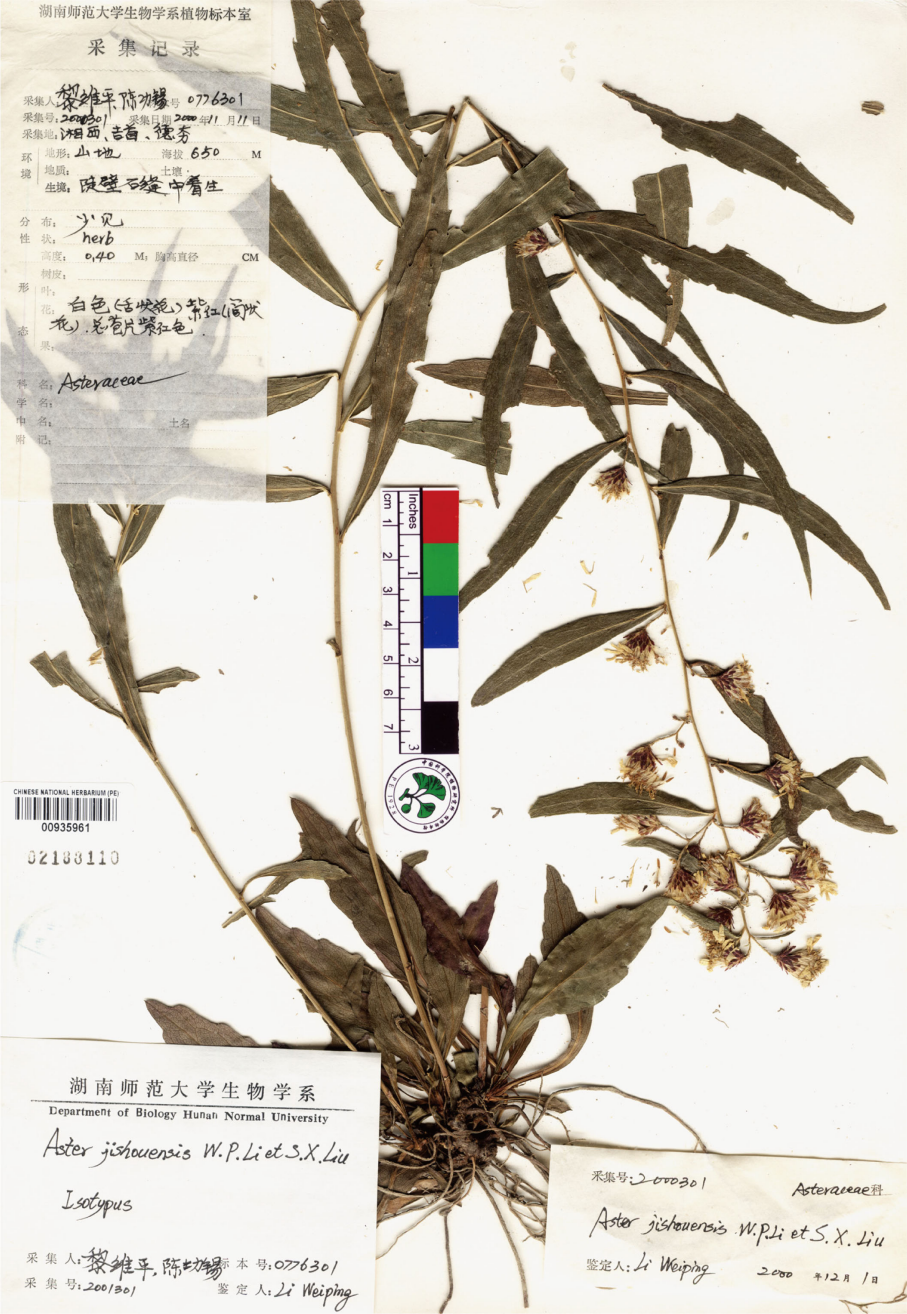
Isotype of *A. jishouensis* W.P. Li and S.X. Liu (voucher, *W.P. Li & G.X. Chen 0776301* (barcode PE 00935961).

**Figure 5:**
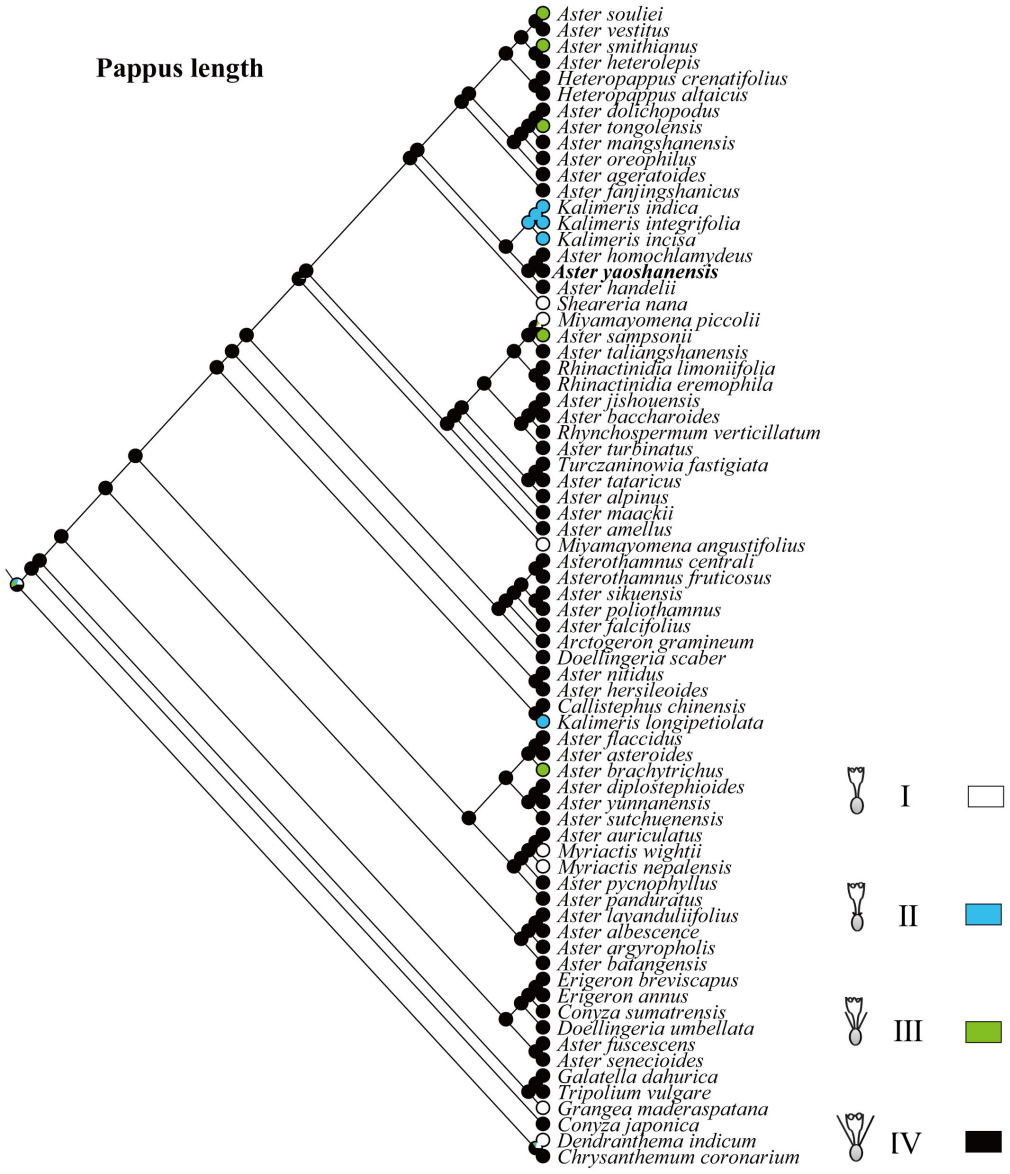
Ancestral character state reconstruction of the length of pappus.

**Figure 6:**
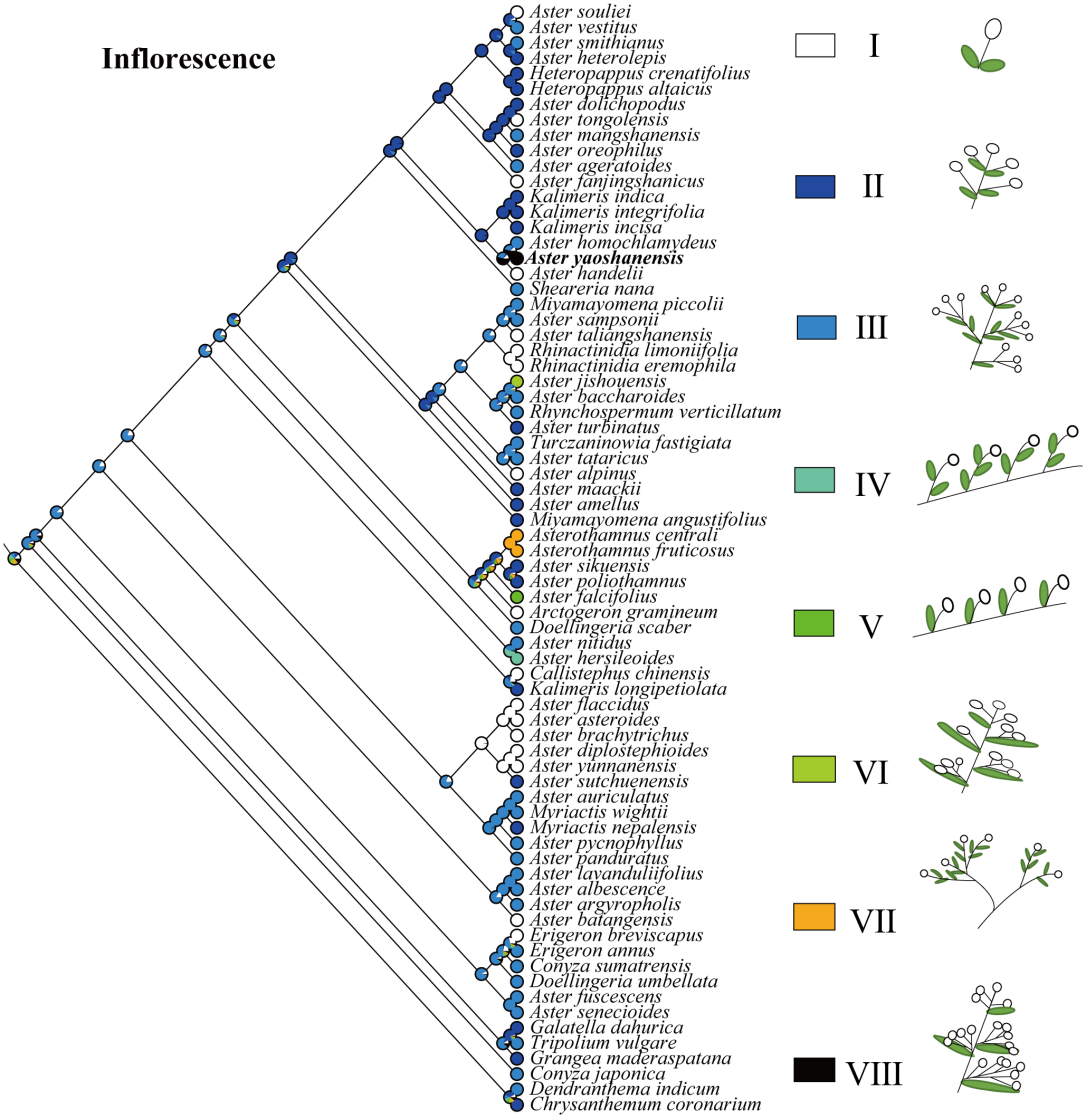
Ancestral character state reconstruction of inflorescence type.

**Figure 7:**
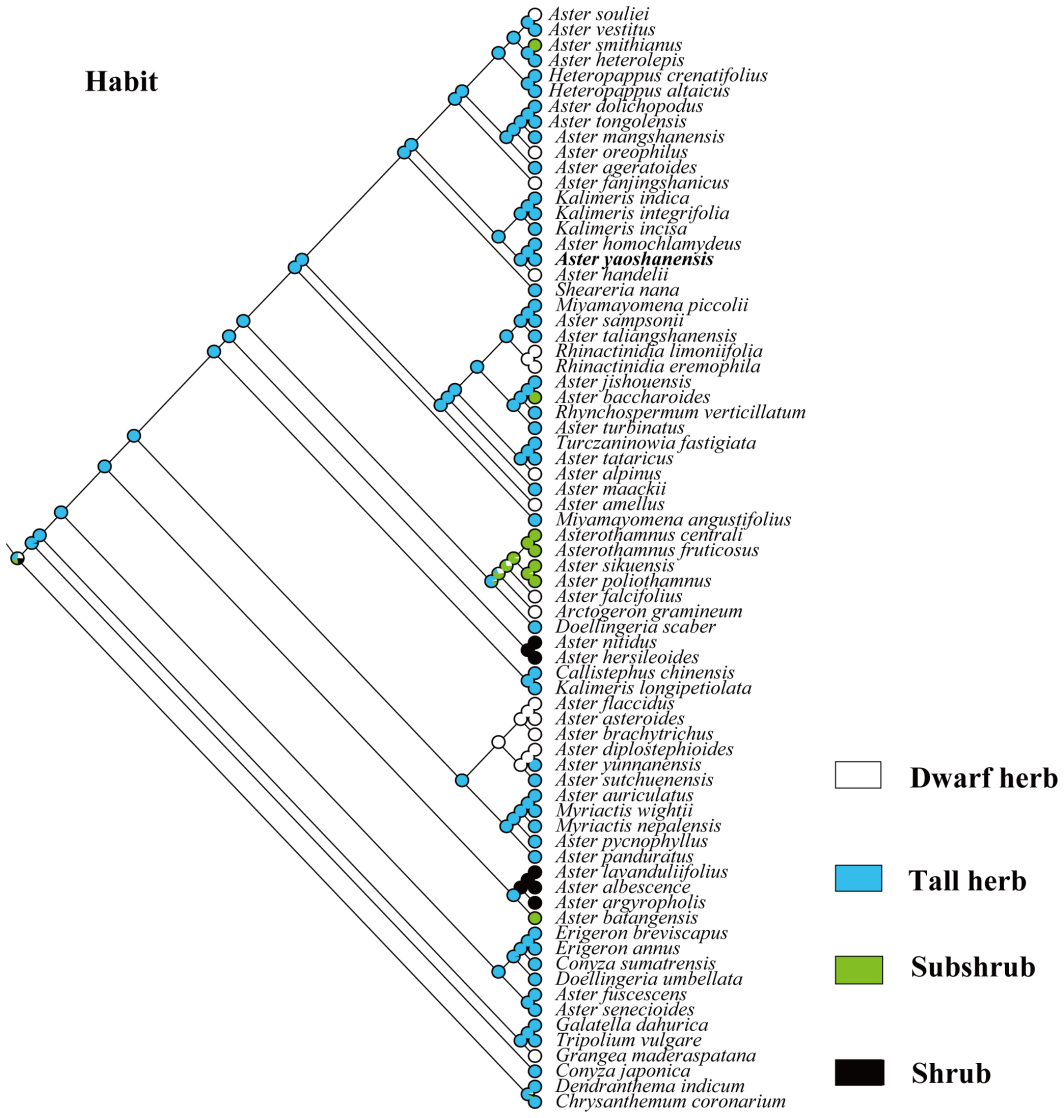
Ancestral character state reconstruction of habit.

Considering the similarity between new species and its related species in *Aster*, we obtained the morphological characteristics of the latter from specimens and literature, and made morphological comparisons between these species (**Table 6**). The new species is most similar to *Aster jishouensis* W.P. Li & S.X. Liu (**Figure 4**). It is mainly distinguished from the latter by its erect (versus oblique ascensional) stem, deep purple (vs. green) basal leaves, 4–5 seriate (vs. 5–7seriate) phyllaries, numerous (vs. 1–4) capitula. In addition, the results of ancestral trait reconstruction were shown in **Figures 5**–**7**.

**Table 6:** Character comparison of *A. yaoshanensis* and its allied taxa.

Character	*A. yaoshanensis*	*A. jishouensis* W. P. Li et S. X. Liu	*A. handelii* Onno	*A. homochlamydeus* Hand.-Mazz.
Stem	Erect or ascending	Caulis adscendens	Erect or ascending	Erect
Length (cm)	30–150	30–100 cm	9–35	20–50
Leaves	Basal and cauline	Basal and cauline	Basal and cauline	Leaves cauline
Shape	Basal leaves ovate-lanceolate; cauline leaves narrowly lanceolate	Basal leaves narrowly elliptical; cauline leaves lineari-lanceolatae usque oblongo-lanceolatae	Basal and lower cauline leaves spatulate to oblanceolate; middle cauline leaves oblong to lanceolate	Ovate to lanceolate
Color	Basal leaves adaxially green, abaxially purplish	/	/	Abaxially pale green, adaxially green
Petiole (cm)	Narrowly winged petiolate	Narrowly winged petiolate	Winged petiolate	Narrowly winged petiolate
Size (cm)	3–15 × 2–4	7–12 × 1.2–2.6	0.7–4 × 0.4–1.4	5–10 × 1.5–4
Indumentum	Adaxially strigillose; abaxially strigillose in the vein	Subglabrous or sparsely glabrous	Both surfaces strigose, sometimes sparsely to moderately minutely stipitate glandular, abaxial veins moderately to densely villous	Abaxially glabrous, veins strigillose to scabridulous, adaxially densely scabridulous
Margins	Margin undulant serrate	Serrate	Margin entire, sometimes sinuate	Coarsely serrate
Capitula	numerous	1–4	1	1–10
Diameter (cm)	1.5–2	/	4–5.5	/
Phyllaries	4–5 seriate	5–7 seriata	2 seriate	2 or 3 seriate
Color	Narrowly green apically	Purpureo-rubra vel apice purpureo-rubra	Tip purplish	Apex acuminate, sometimes purplish
Size (mm)	Outer phyllaries 1.5–2 × ca.1 mm, Inner phyllaries 1–1.5 × 6–7 mm	Outer phyllaries 5–6 × 0.7–1.3 mm Inner phyllaries 1.3–1.5 × ca. 1 mm	7.5–9 × 1.2–2	Outer phyllaries 6–8 × ca. 1.5 mm Inner phyllaries 5–9 × ca. 2 mm
Ray florets color	White	White	Bluish purple to lavender blue	White or purple
Ray florets lamina size (mm)	6–7 × 1–1.5	7–9.6 × 1.5–2.3	12–23 × 2.5–3	10–15 × 2–3
Distribution	1100 m, Guangxi	650 m, Hunan	3000–3500 m. SW Sichuan, NW Yunnan.	3000–3700 m. S Gansu, SW Sichuan, NW Yunnan.

### 3.5 Taxonomic treatment

**Figure 8:**
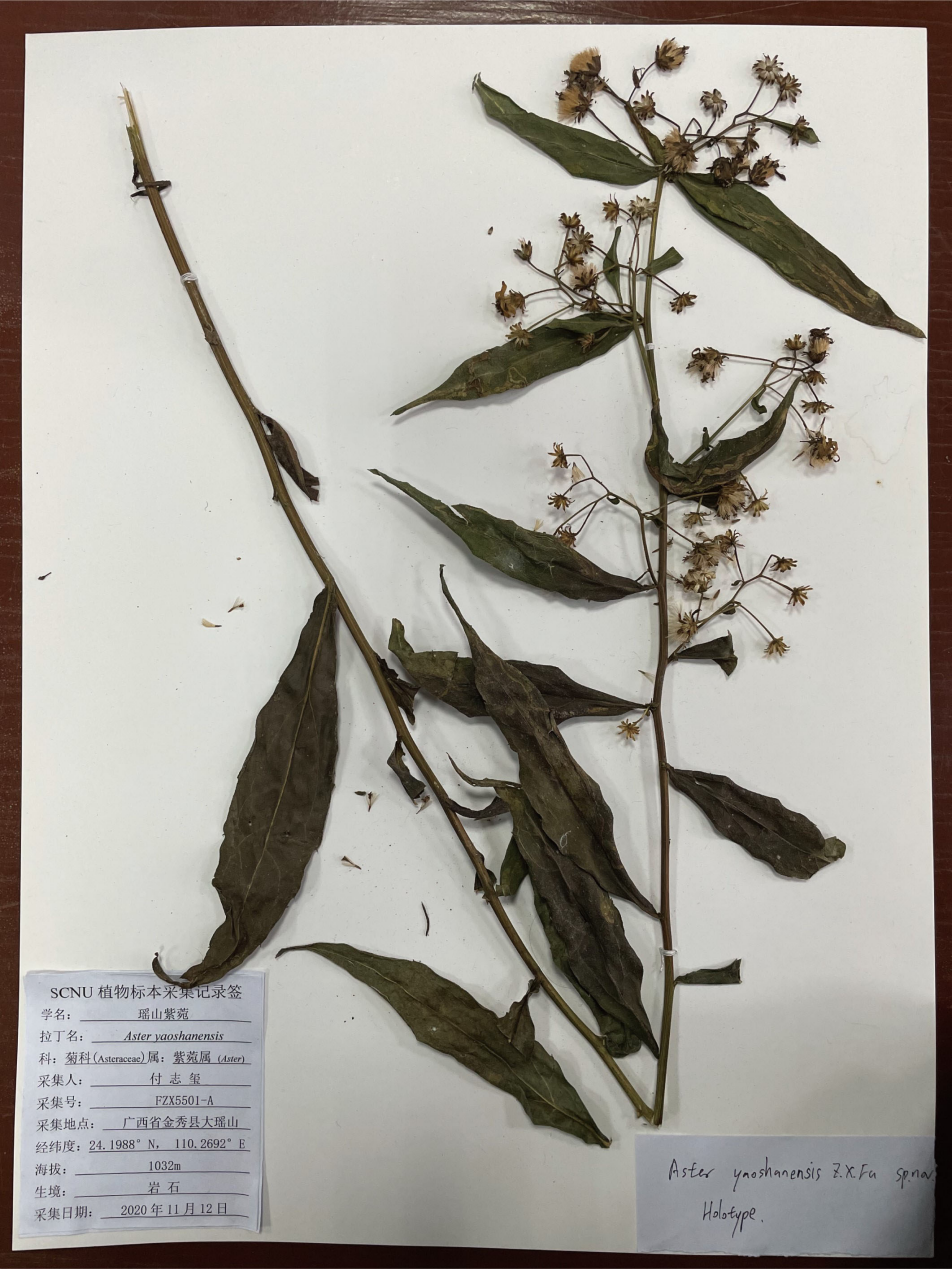
Holotype of *A. yaoshanensis* sp. nov. K. Qin, Z.X. Fu and P. Li. (Upper part) (voucher, *Z.X. Fu 5501*, SCNU).

**Figure 9:**
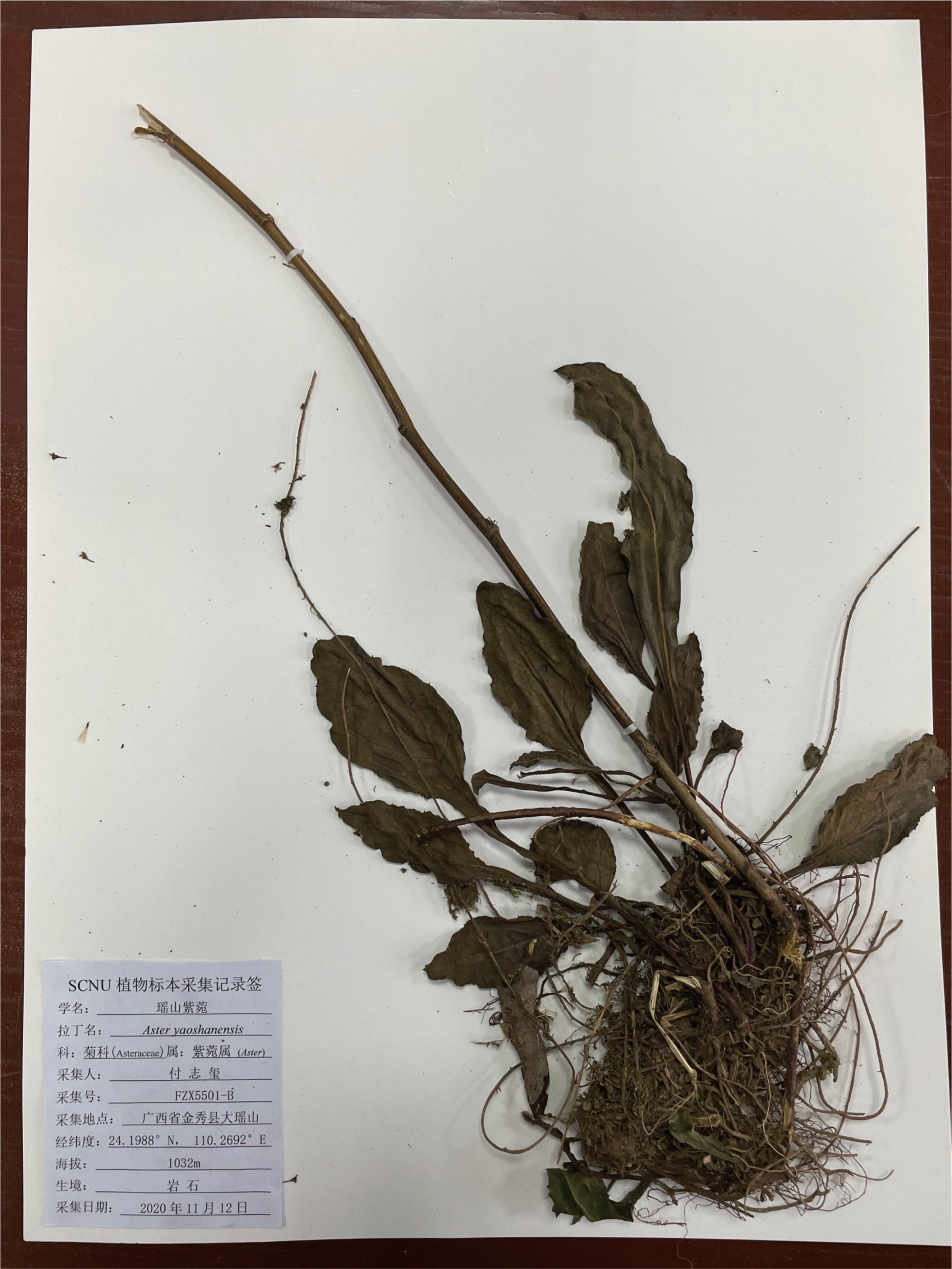
Holotype of *A. yaoshanensis* sp. nov. K. Qin, Z.X. Fu and P. Li. (Lower part) (voucher, *Z.X. Fu 5501*, SCNU).

***Aster yaoshanensis*** K. Qin, Z.X. Fu & P. Li, sp. nov. (**Figures 8**, **9**)

**Type (Holotype).** China, Guangxi, Laibin city, Jinxiu county, Dayaoshan Mountain National Nature Reserve, ca. 1100 m a.s.l., 24.1988°N, 110.2692°E, limestone, 12 November 2020, Zhixi Fu FZX5501 (SCNU).

**Figure 10:**
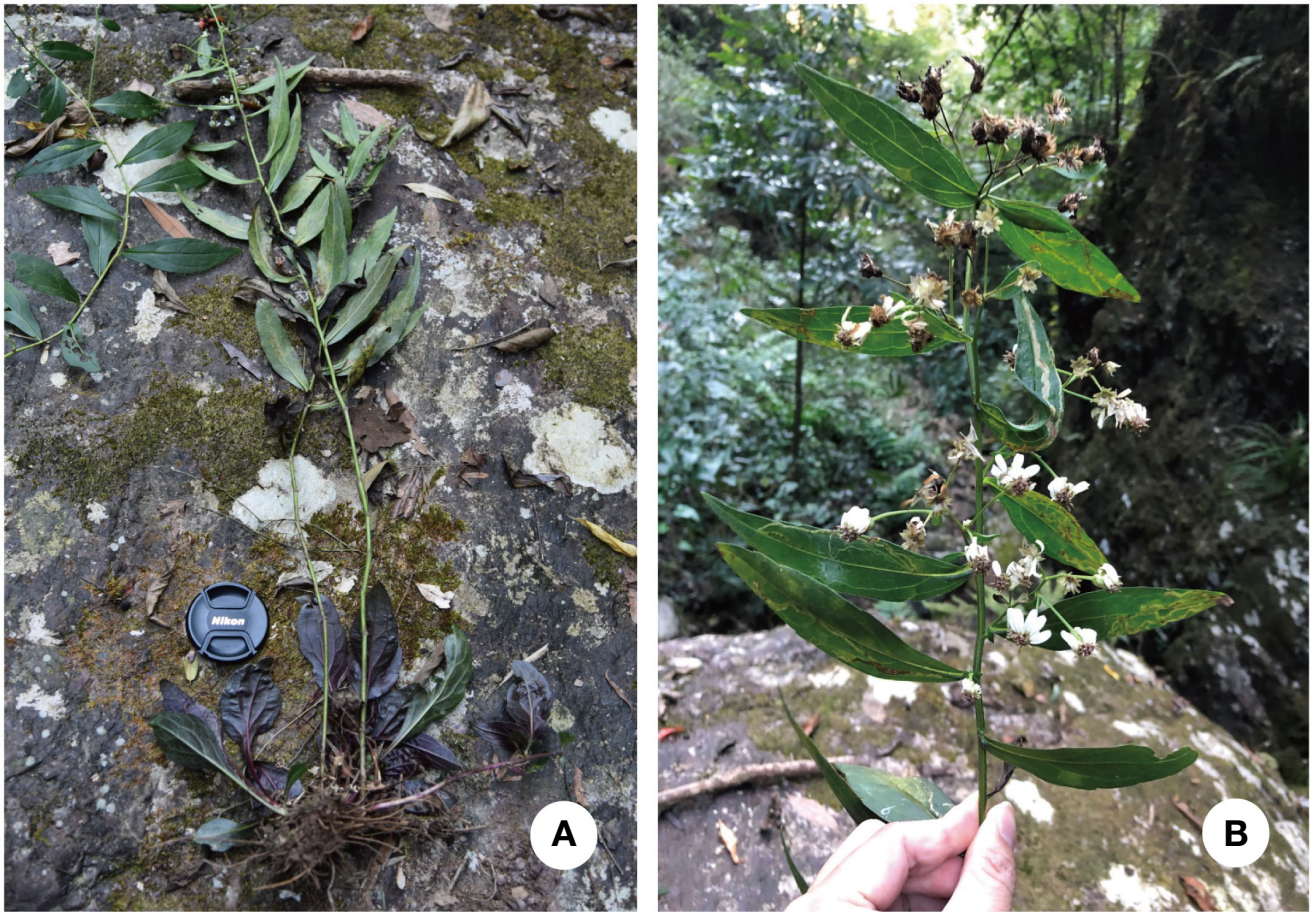
*A. yaoshanensis* K. Qin, Z.X. Fu and P. Li (voucher, *Z.X. Fu 5501*, SCNU): **(A)** Habit; **(B)** Inflorescences, showing capitula terminal on lateral branches.

**Figure 11:**
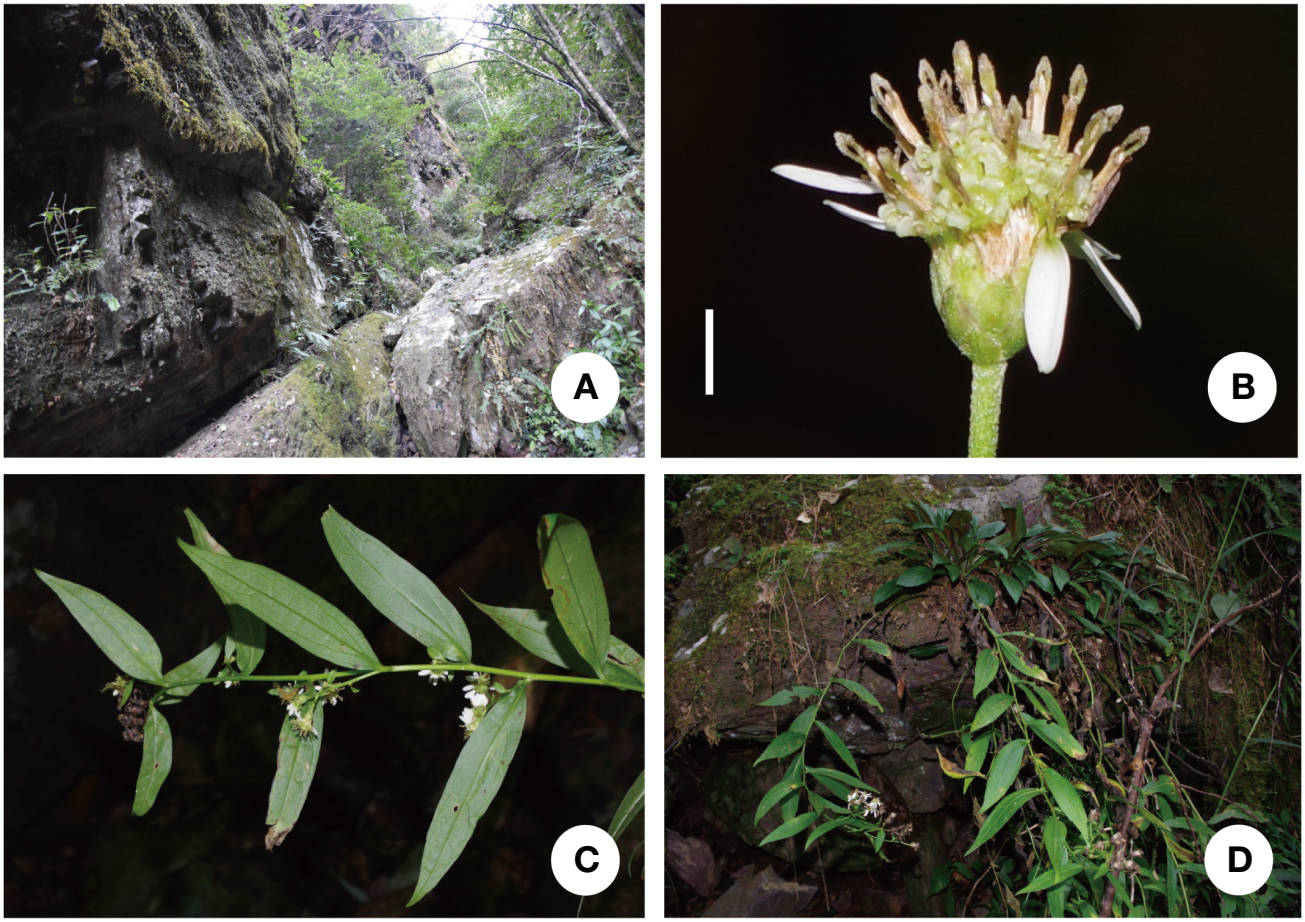
*A. yaoshanensis* (voucher, *Z. X. Fu 5501*, SCNU): **(A)** Habitat; **(B)** Capitulum, lateral view, showing the involucre. **(C)** Abaxial surface of leaf blade; **(D)** Adaxial surface of leaf blade. Scale Bar: 2 cm.

**Description.** Perennial herbs, 90–130 cm tall, basally woody, rhizomatous. Stems erect, unbranched, finely striate, smooth and glabrous with many basal leaves and some cauline leaves. Basal leaves rather thick, ovate-lanceolate, apex acute to slightly obtuse, petiole narrowly winged, 3–3.5 cm × 1–1.5 cm, margin undulant serrate, abruptly mucronate, adaxially green, strigillose; abaxially amaranth, the vein strigillose. Cauline leaves, slightly reduced downward, adaxially green, densely strigose; abaxially subglabrous, midvein prominent abaxially, lowest leaves withered by anthesis, lower to upper leaves sessile or shortly petiole, the uppermost very small, blade narrowly lanceolate to oblanceolate, 2–14 cm × 1–4 cm, margin entire to undulant serrate (teeth mucronulate). Flowering branches attach to the leaf-axils, capitula numerous, in terminal or axillary radialized, 1.5–2 cm in diam; peduncles 2–8 mm, smooth and glabrous, bracts ovate, margin entire. Involucres campanulate, 6–7 mm × 2–3 mm, phyllaries 4-5 seriate, unequal, narrowly green apically, abaxially moderately to densely strigose, margin scarious, apex acute to obtuse, ciliate, outer phyllaries ovate, 1.5–2 × ca. 1 mm, middle phyllaries lanceolate-oblong, 2–2.5 × ca. 1 mm, inner phyllaries approximately oblong, 3–3.5 × 1–1.5 mm. Ray florets 8-18, white, lamina 6–7 mm × 1–1.5 mm, glabrous, eglandular; disk florets yellowish-green, 5–6 mm, tube base flared, lobes capreolary, tip recurved to cannular. Achenes long-obovoid, compressed, ca. 1.8–2.2 mm, 2-3-ribbed, sparsely strigillose. Pappus, bristles 2.5–3.5 mm. (**Figures 10**, **11**).

**Etymology.** The specific epithet refers the famous Dayaoshan Mountain in Jinxiu county, Laibin city, Guangxi autonomous region, China.

**Figure 12:**
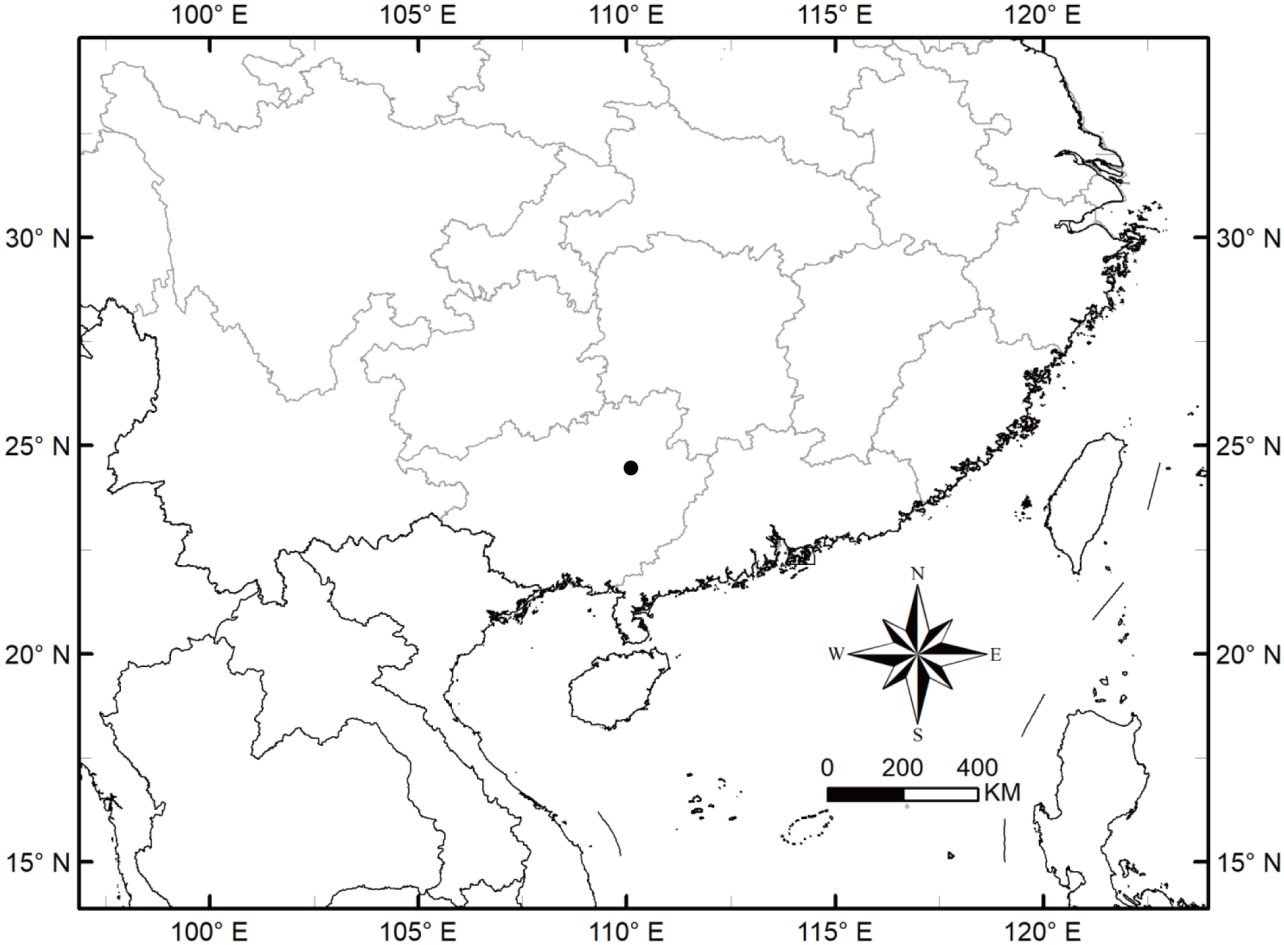
Distribution map of *A. yaoshanensis* (●).

**Distribution and habitat.** The new species, *A. yaoshanensis* is mainly distributed at an altitude of about 1000 m in Dayaoshan Mountain, Guangxi (**Figure 12**), and mainly growing on limestone, slopes, forest margins, flooded lands, along streams, wet places.

**Phenology.** Flowering and fruiting from September to November.

**Conservation status**. According to the IUCN, 2024 categories and criteria, newly described species with a restricted distribution area are often considered in a threat category (CR, EN, VU) (Zhang et al., 2019; Blasco et al., 2021; Wagensommer and Venanzoni, 2021; Ibáñez et al., 2022), but more exploration in the occurring area of the new species is necessary for defining the distribution, population size, threats, etc., before being able to assess the conservation status of *A. yaoshanensis*. The new species is currently only found in rock crevices of mountain valleys in Dayaoshan National Nature Reserve. It is a narrowly distributed species with no more than 30 individuals found. It is very possible that the species is found in other localities with a more comprehensive collecting programme. Therefore, it would be best to assess the conservation status of the species as DD (Data Deficient).

**Additional specimens examined (Paratypes).** CHINA. Guangxi: Jinxiu county, Dayaoshan Mountain National Nature Reserve, alt. 1100m a.s.l., limestone, 12 November 2020, *FZX5505* (SCNU), *FZX5508* (SCNU), *FZX5509* (SCNU) *FZX5510* (SCNU), and *FZX5511* (SCNU).

## 4 Discussion

### 4.1 Plastome structure and characteristics analysis

In our study, we successfully assembled the complete plastome of *A. yaoshanensis* collected from Guangxi for the first time. Based on the analysis of the plastome map, we found that the structure, gene location, size and gene content of *A. yaoshanensis* are highly conserved, which is very similar to the plastome structure of most reported *Aster* plants, such as *A. ageratoides* (Feng et al., 2021; Zhang et al., 2021; Wang and Liu, 2023). The plastomes of these *Aster* have a standard quadripartite structure, including two inverted repeat regions (IRs), an LSC region and an SSC region, and the general GC content was low in the whole sequence. This indicates the close relationship between *A. yaoshanensis* and *Aster*.

### 4.2 Phylogenetic analysis of the ITS data set

In order to further determine the evolutionary position of *A. yaoshanensis* in *Aster*, we conducted phylogenetic analysis of ITS sequences of *Aster* and its related genera (**Figure 2**). *Heteropappus*, *Kalimeris*, *Sheareria*, *Rhynchospermum*, *Rhinactinidia*, *Miyamayomena*, *Turczaninowia*, *Doellingeria*, and other *Aster* taxa were deeply nested within the core *Aster* clade; this is consistent with previous studies (Li et al., 2012). In the *Aster* clade*, A. yaoshanensis* formed a compact clade with *A. homochlamydeus* and *A. handelii* (PP = 1.00, BS = 100), and the clade was sister to the clades comprising some *Aster* taxa, *Heteropappus* and *Kalimeris*. The result further proved that *A. yaoshanensis* is closely related to *Aster*. In addition, *A. jishouensis* and *A. yaoshanensis* were apparently embedded in different clades, indicating that the two species are not closely related.

### 4.3 Phylogenetic analysis of the complete plastome sequence data set

Phylogenetic analysis of 17 plastomes within *Aster* and its related genera revealed that *A. yaoshanensis* is sister to *A. ageratoides*, and they form a powerful clade (BS = 100 in **Figure 3**), which was sister clade to the clade comprising with six *Aster* species (BS = 100). At the same time, *Heteroplexis* is not monophyletic and embedded within the *Aster* clade, which is sister clade to above eight *Aster* species (including *A. yaoshanensis*, BS = 100). This is consistent with Duan’s findings (Duan et al., 2022). Therefore, the *Aster* clade had strong support, and *A. yaoshanensis* should be merged into *Aster.*

### 4.4 Ancestral trait reconstruction

Pappus length, inflorescence, and habit were often considered to be important diagnostic characters in previous taxonomic treatments of *Aster* and its related genera (Li et al., 2012; Norbert et al., 2017).

Pappus length. In Asteraceae, sepals usually degenerate to pappus to help the dispersal of fruits or seeds, and the pappus of *Aster* plants are varied in morphology (Chen et al., 2011; Norbert et al., 2017). Therefore, it is one of the important features in the classification of *Aster* and its related genera (Chen, 1986; Li et al., 2012). Based on the length of pappus, these plants are divide into four types (I–IV): none pappus, short pappus, long pappus, and longer pappus. The result indicated that type IV with a probability of 98% was the common ancestral trait (**Figure 5**). *A. yaoshanensis* have longer pappus, belonging to the typical type IV.

Inflorescence. In genus *Aster*, the inflorescences belong to capitula (Chen et al., 2011), and the evolution of inflorescence is diverse (Li et al., 2012). Based on the number of capitulum and the way of inflorescences branching, *Aster* and its related genera could be divided into seven types (I–VII), and the legend can be seen in **Figure 6** for details. The type of inflorescence is one of the important identification morphological characters of *A. yaoshanensis* as a new species, we define it as Type VII. Compared with *A. handelii* (I) and *A. homochlamydeus* (III) of the same subclade, *A. yaoshanensis* has more numbers of capitula, which mostly grows in the branch ends and leaf axils. *A. jishouensis* (VI) is very similar to *A. yaoshanensis*, but inflorescences have only 1–4 capitula. In addition, the ancestral reconstruction result showed that type I evolved independently at least 10 times in this taxa, type II originated from type III, and the evolutionary transformation may have occurred more than once.

Habit. In our analysis, we divided the habit of *Aster* and its relatives into dwarf herb, tall herb, subshrub, and shrub (Chen et al., 2011; Norbert et al., 2017; Jabbour et al., 2018). Phylogenetic analysis confirmed that tall herbs were the most probable ancestral feature of these plants (96%, **Figure 7**). Like *A. jishouensis*, *A. yaoshanensis* belongs to the tall herb, which may be the result of the evolution of the two plants to adapt to similar habitats. In addition, we found that dwarf herbs evolved independently at least nine times, subshrubs four times, and shrubs only twice.

## 5 Conclusion

Based on the multi-methods analysis, *A. yaoshanensis* is indicated as a new species. We assembled and analyzed the whole plastome analysis and understood the structural characteristics of the whole plastome. Phylogenetic analyses of ITS sequence with 71 species of *Aster* and its related genera and the whole plastome with 17 species of *Aster* further revealed the evolutionary relationship of *A. yaoshanensis*. The results showed that *A. yaoshanensis* is located in the *Aster* clade. Results of morphological comparison and ancestral traits reconstruction showed that *A. yaoshanensis* and its some morphological related *Aster* spp., especially *A. jishouensis*, are similar, but there are some significant differences. For example, the former has more capitula.

The results of this study can provide references for future studies on species identification, phylogeny and characteristic evolution of *Aster*. At the same time, the analysis of phylogeny, morphological comparison and ancestral reconstruction in this study still have some limitations. In future studies, we need to expand the sample size and obtain more morphological data to increase the availability of data and more comprehensively analyze and discuss the phylogenetic and evolutionary characteristics of *Aster*.

## Data availability statement

The datasets presented in this study can be found in online repositories. The names of the repository/repositories and accession number(s) can be found below: https://www.ncbi.nlm.nih.gov/, OL461705.1 https://www.ncbi.nlm.nih.gov/, ON120847.1.

## Author contributions

XZ: Conceptualization, Writing – original draft. KQ: Investigation, Writing – review & editing. TL: Data curation, Methodology, Writing – review & editing. TQ: Data curation, Methodology, Writing – review & editing. JL: Data curation, Methodology, Writing – review & editing. GZ: Conceptualization, Writing – original draft. BL: Conceptualization, Writing – original draft. PL: Supervision, Writing – review & editing. ZF: Investigation, Supervision, Writing – review & editing.
